# Supplementary material for: Targeted genome editing by lentiviral protein transduction of zinc-finger and TAL-effector nucleases
Source: eLife. 2014 Apr 24;3:e01911. doi: 10.7554/eLife.01911 (PMC3996624; doi:10.7554/eLife.01911)
Supplement: Table 1—source data 1. — Genomic DNA of HEK293-eGFPmut reporter cells transduced with 600 ng p24 LP-ZFNLR(gfp) was used as PCR template for amplification and subsequent cloning of the part of the egfp gene encompassing the region recognized by the two ZFNs. The wild-type sequence is shown at the top. The net change of length caused by indels is indicated to the right of each sequence. Green dashes represent deleted nucleotides, red lower case letters represent nucleotide substitutions, whereas blue lower case letters illustrate inserted nucleotides. If one particular sequence appeared in more than one clone, the exact number of clones with this particular sequence is provided in parenthesis. DOI: http://dx.doi.org/10.7554/eLife.01911.006 [file elife01911s001.pdf]

Mutations in HEK293-eGFPmut reporter cells induced by LP-ZFNLR(gfp)

GCGTGCAGTGCTTCAGCCGCTACCCCTAACATGAAGCAGCACGACTTCTTCAAGTCCG	WT
GCGTGCAGTGCTTCAGCCGCTACCCCTAACA----GCAGCACGACTTCTTCAAGTCCG	-4
GCGT--TCTTCAAGTCCG	-42
GCGTGCAGTGCTTCAGCCGCTAC----------------------TTCTTCAAGTCCG	-22
GCGTGCAGTGCTcCAGCCGCTACCCCTAACATGAAGCAGCACGACTTCTTCAAGTCCG	0
GCGTGCAGTGCTTCAGCCGCTACCCCTAAaCATGAAGCAGCACGACTTCTTCAAGTCC	+1
GCGTGCAGTGCTTCAGCCGCTACCCCTAACAaacaTGAAGCAGCACGACTTCTTCAAG	+4 (×3)
