# Supplementary material for: Targeted genome editing by lentiviral protein transduction of zinc-finger and TAL-effector nucleases
Source: eLife. 2014 Apr 24;3:e01911. doi: 10.7554/eLife.01911 (PMC3996624; doi:10.7554/eLife.01911)
Supplement: Table 1—source data 2. — Genomic DNA of cells transduced with 200 ng p24 LP-ZFNLR(CCR5) was used as PCR template for amplification and subsequent cloning of a CCR5 amplicon encompassing the region recognized by the two ZFNs. The wild-type sequence is shown at the top. Types of indels are indicated as described in the legend to Table 1—source data 1. DOI: http://dx.doi.org/10.7554/eLife.01911.007 [file elife01911s002.pdf]

CCR5 mutations in HEK293 induced by LP-ZFNLR(CCR5)

```
TTGGTTTTGTGGGCAACATGCTGGTCATCCTCATCCTGATAAACTGCAAAAGGCTGAAGAGCATGACTGACATCT   WT
TTGGTTTTGTGGGCAACATGCTGGTCATCCTCATC------------AAAAGGCTGAAGAGCATGACTGACATCT   -12
TTGGTTTTGTGGGCAACATGCTGGTCATCC-----CTGATAAACTGCAAAAGGCTGAAGAGCATGACTGACATCT   -5
TTGGTTTTGTGGGCAACATGCTGGTCATCCTCAT------AAACTGCAAAAGGCTGAAGAGCATGACTGACATCT   -6
TTGGTTTTGTGGGCAACATGCTGGTCATCCTCATCCTGATAAACTGCAgAAGGCTGAAGAGCATGACTGACATCT   0
TTGGTTTTGTGGGCAACATGCTGGTCATCCTCATCCTGATccACTGCAAAAGGCTGAAGAGCATGACTGACATCT   0
TTGGTTTTGTGGGCAACATGCTGGTCATCCTCATCCTGATtAAACTGCAAAAGGCTGAAGAGCATGACTGACATC   +1
TTGGTTTTGTGGGCAACATGCTGGTCATCCTCATCCTGATtgatAAACTGCAAAAGGCTGAAGAGCATGACTGAC   +4
```

CCR5 mutations in NHDFs induced by LP-ZFNLR(CCR5)

```
TTGGTTTTGTGGGCAACATGCTGGTCATCCTCATCCTGATAAACTGCAAAAGGCTGAAGAGCATGACTGACATCT   WT
TTGGTTTTGTGGGCAACATGCTGGTCATCCTCA-------AAACTGCAAAAGGCTGAAGAGCATGACTGACATCT   -7
TTGGTTTTGTGGGCAACATGCTGGTCATCCTCA---------------AAAGGCTGAAGAGCATGACTGACATCT   -15
TTGGTTTTGTGGGCAACATGCTGGTCATCCTCATC-----AAACTGCAAAAGGCTGAAGAGCATGACTGACATCT   -5
TTGGTTTTGTGGGCAACATGCTGGTCATCCTCATC---------TGCAAAAGGCTGAAGAGCATGACTGACATCT   -9
TTGGTTTTGTGGGCAACATGCTGGTCATCCTCATCTtAatAAACTGCAAAAaGCTGAAGAGCATGACTGACATCT   0
TTGGTTTTGTGGGCAACATGCTGGTCATCCTCATCCT--cAAACTGCAAAAGGCTGAAGAGCATGACTGACATCT   -2
TTGGTTTTGTGGGCAACATGCTGGTCATCCTCATCCTGATctgatAAACTGCAAAAGGCTGAAGAGCATGACTGA   +5
```

CCR5 mutations in HKs induced by LP-ZFNLR(CCR5)

```
TTGGTTTTGTGGGCAACATGCTGGTCATCCTCATCCTGATAAACTGCAAAAGGCTGAAGAGCATGACTGACATCT   WT
TTGGTTTTGTGGGCAACATGCTGGTCATCC-----CTGATAAACTGCAAAAGGCTGAAGAGCATGACTGACATCT   -5
TTGGTTTTGTGGGCAACATGCTGGTCATCCTCATCCTGAT-----GCAAAAGGCTGAAGAGCATGACTGACATCT   -5
TTGGTTTTGTGGGCAACATGCTGGTCATCCTCATC---------TGCAAAAGGCTGAAGAGCATGACTGACATCT   -9
TTGGTTTTGTGGGCAACATGCTGGTCATCCTCATC-----AAACTGCAAAAGGCTGAAGAGCATGACTGACATCT   -5
TTGGTTTTGTGGGCAACA---------------------------GCAAAAGGCTGAAGAGCATGACTGACATCT   -27
TTGGTTTTGTGGGCAAC-------------------------------AAAGGCTGAAGAGCATGACTGACATCT   -31
TTGGTTTTGTGGGCAACATGCTGGTC----------TGATAAACTGCAAAAGGCTGAAGAGCATGACTGACATCT   -10
TTGGTTTTGTGGGCAACATGCTGGTCATCCTCATCCTGATAAA-------AGGCTGAAGAGCATGACTGACATCT   -7
TTGGTTTTGTGGGCAACATGCTGGTCATCCTCATCtTtAaAAACTGCAAAAaGCTGAAGAGCATGACTGACATCT   0
TTGGTTTTGTGGGCAACATGCTGGT------------AATAAACTGCAAAAaGCTGAAGtGCtTGACTGACATtT   -12
TTGGTTTTGTGGGCAACATGCTGGTCATCCTCATCCTGATctgatAAACTGCAAAAGGCTGAAGAGCATGACTGA   +5
```
