# Supplementary material for: Targeted genome editing by lentiviral protein transduction of zinc-finger and TAL-effector nucleases
Source: eLife. 2014 Apr 24;3:e01911. doi: 10.7554/eLife.01911 (PMC3996624; doi:10.7554/eLife.01911)
Supplement: Table 1—source data 3. — Genomic DNA of cells transduced with 200 ng p24 LP-ZFNLR(AAVS1) was used as PCR template for amplification and subsequent cloning of an AAVS1 amplicon encompassing the region recognized by the two ZFNs. The wild-type sequence is shown at the top. Types of indels are indicated as described in the legend to Table 1—source data 1. If one particular sequence appeared in more than one clone, the exact number of clones with this sequence is provided in parenthesis. DOI: http://dx.doi.org/10.7554/eLife.01911.008 [file elife01911s003.pdf]

AAVS1 mutations in HEK293 induced by LP-ZFNLR(AAVS1)

GGGTACTTTTATCTGTCCCCTCCACCCCACAGTGGGGCCACTAGGGACAGGATTGGTGACAGAAAAGCCCCATCC	WT
GGGTACTTTTATCTGTCCCCTCCACCC---------GCCACTAGGGACAGGATTGGTGACAGAAAAGCCCCATCC	-9
GGGTACTTTTATCTGTCCCCTCCACCCCA--------------------GGATTGGTGACAGAAAAGCCCCATCC	-20 (×2)
GGGTACTTTTATCTGTCCCCTCCACCCCACAGTGG-----------ACAGGATTGGTGACAGAAAAGCCCCATCC	-11
GGGTACTTTTATCTG--------ACCCCACAGTGGGGCCACTAGGGACAGGATTGGTGACAGAAAAGCCCCATCC	-8
GGGTACTTTTATCTGTCCCCTCCACCCCACAGTGGGGCCAGGTAACCAGCTTGGCCAggtaaccagcttggccag<br>gtgacaccgcagtggtgaggaaggggaggccactaggcccagctgcgccaCTAGGGACAGGATTGGTGACAGAAA	+68

AAVS1 mutations in NHDFs induced by LP-ZFNLR(AAVS1)

GGGTACTTTTATCTGTCCCCTCCACCCCACAGTGGGGCCACTAGGGACAGGATTGGTGACAGAAAAGCCCCATCC	WT
GGGTACTTTTATCTGTCCCCTCCACCCCACAGTGG-----------ACAGGATTGGTGACAGAAAAGCCCCATCC	-11
GGGTACTTTTATCTGTCCCCTCCACCCCACAGTGGG----------ACAGGATTGGTGACAGAAAAGCCCCATCC	-10 (×3)
GGGTACTTTTATCTGTCCCCTCCACCCCACAGTGGGGCCA------ACAGGATTGGTGACAGAAAAGCCCCATCC	-6
GGGTACTTTTATCTGTCCCCTCCACCCCACAGT----------GGGACAGGATTGGTGACAGAAAAGCCCCATCC	-10
GGGTACTTTTATCTGTCCCCTCCACCCCACAGTGGGGCCAgccaCTAGGGACAGGATTGGTGACAGAAAAGCCCC	+4

AAVS1 mutations in HKs induced by LP-ZFNLR(AAVS1)

GGGTACTTTTATCTGTCCCCTCCACCCCACAGTGGGGCCACTAGGGACAGGATTGGTGACAGAAAAGCCCCATCC	WT
GGGTACTTTTATCTGTCCCCTCCACCCCACAGTGGG----------ACAGGATTGGTGACAGAAAAGCCCCATCC	-10 (×2)
GGGTACTTTTATCTGTCCCCTCCACCCCACAG------------GGACAGGATTGGTGACAGAAAAGCCCCATCC	-12 (×2)
GGGTACTTTTATCTGTCCCCTCCACCCCACAGTGGG----CTAGGGACAGGATTGGTGACAGAAAAGCCCCATCC	-4
GGGTACTTTTATCTGTCCCCTCCACCCCAC------------AGGGACAGGATTGGTGACAGAAAAGCCCCATCC	-12 (×2)
GGGTACTTTTATCTGTCCCCTCCACCCCACA----------TAGGGACAGGATTGGTGACAGAAAAGCCCCATCC	-10
