# Supplementary material for: Targeted genome editing by lentiviral protein transduction of zinc-finger and TAL-effector nucleases
Source: eLife. 2014 Apr 24;3:e01911. doi: 10.7554/eLife.01911 (PMC3996624; doi:10.7554/eLife.01911)
Supplement: Supplementary file 1. — Oligonucleotides used in this study, ‘Targeted genome editing by lentiviral protein transduction of zinc-finger and TAL-effector nucleases’. DOI: http://dx.doi.org/10.7554/eLife.01911.014 [file elife01911s004.docx]

**Supplementary file 1**

**Oligonucleotides used in Cai *et al.*, ‘Targeted genome editing by lentiviral protein transduction of zinc-finger and TAL-effector nucleases’**

| 4684 | ATTGACGCAAATGGGCGGTAG |
| --- | --- |
| BGHpA | TCCCCAGCATGCCTGCTATT |
| YJ001R | ATGTTAGGGGTAGCGGCTGAAGCACT |
| YJ002F | TCAGCCGCTACCCCTAACATGAAGCAGCACGACTTCTTCA |
| YJ003F | TCAGGGGCCCTGCCCATCCTGGTCGAGCTG |
| YJ004R | TGCACTCGAGTTATCGGGAGCGGCGATACCGTA |
| YJ112F | TGACCCCGGGCGCCACCATGGCCCCCAAGAAGAAGAG |
| YJ113R | TGACCCCGGGCTGCACAATCGGATAGTTCTGGCTAAAGTTTATCTCGCCGTTAT |
| YJ150F | TCAGCTCGAGTGCCCATCCTGGTCGAGCTG |
| YJ151R | TGCAGGGCCCTTATCGGGAGCGGCGATACC |
| YJ168F | TGACCCCGGGCGCCACCATGTGCTATCCGTATGATGTGCCGGATTATGCGAGCCTGGCCCCCAAGAAGAAGAGGAA |
| YJ170R | TTACTTGTACAGCTCGTCCA |
| YJ175F | TGACCCCGGGCGCCACCATGGCTCCCAAAAAGAAAAG |
| YJ176F | TGACCCCGGGCGCCACCATGGCTCCTAAAAAGAAAAG |
| YJ177F | TGACCCCGGGCGCCACCATGGATTACAAAGACCACGA |
| YJ180F | TGACGAATTCTGCGGTTTTGGCAGTACATCAATGG |
| YJ181R | TGACGGATCCTTACTTGTACAGCTCGTCCATGCCG |
| YJ186R | TGACCCCGGGCTGCACAATCGGATAGTTCTGGCTAAAGTTTATCTCACCGTTAT |
| YJ191F | ACCGGTTCTAGACGTACGGTCGAC |
| YJ193F | TGACGGGCCCGCCTGGGCGACAGAGTGAGACCCTG |
| YJ195F | GTTCTAGACGTACGGTCGACGCTGAAGAGCATGACTGACATCTAC |
| YJ196R | TGACCTCGAGAACAATCATGATGGTGAAGATAAGC |
| YJ200F | TGACACGCGTTCCTTCTCGGCGCTGCACCACGTG |
| YJ201R | GTCGACCGTACGTCTAGAACCGGTGGAGGGGACAGATAAAAGTACCCAG |
| YJ202F | GTTCTAGACGTACGGTCGACTGGTGACAGAAAAGCCCCATCCTT |
| YJ203R | TGACCTCGAGAAGAGAAAGGGAGTAGAGGCGGCC |
| YJ205R | AGATAGCACTGGGGACTCTTTAAG |
| YJ206R | GTCGACCGTACGTCTAGAACCGGTCAGCATGTTGCCCACAAAACCAAAG |
| YJ207F | AGCAAACCTTCCCTTCACTACAAA |
| YJ208R | AAGGTGTTCAGGAGAAGGACAATG |
| YJ211F | TGACCTTAAGGCCACCATGTGCTATCCGTATGATGT |
| YJ212R | TGACCTCGAGTTAAAAGTTTATCTCGCCGTTAT |
| YJ213F | TGACACTAGTACCGGTGCCACCATGGCTTCCTCCCCTCCAAA |
| YJ218F | TGACACTAGTACCGGTGCCACCATGTGCTATCCGTATGATGTGCCGGATTATGCGAGCCTGGCTTCCTCCCCTCCAAAGAA |
| YJ220F | CCACATCTCGTTCTCGGTTT |
| YJ222F | TTCGGGTCACCTCTCACTCC |
| YJ223R | GGCTCCATCGTAAGCAAACC |
| YJ224F | AAAACAGTTTGCATTCATGGAGGGC |
| YJ225R | AGAAGCCTATAAAATAGAGCCCTGT |
| YJ226F | CGTTGTAAAACGACGGCCAG |
| YJ237F | TGACGAATTCAGCAAACCTTCCCTTCACTACAAA |
| YJ238R | TGACGGATCCAAGGTGTTCAGGAGAAGGACAATG |
| YJ240F | TGACGAATTCTTCGGGTCACCTCTCACTCC |
| YJ244R | TAGCAGATCTGGCTCCATCGTAAGCAAACC |
| YJ256F | TGACTCCGGAGCCACCATGTGCTATCCGTA |
| YJ257R | TGACTCCGGACTGCACAATCGGATAGTTCT |
| YJ359R | AATCTTCTTTCTGGCATTTAGTAAA |
| YJ553F | TGACCTTAAGGCCACCATGTGCTATCCGTATGATGT |
| YJ554R | TGACGTCGACTTAAAAGTTTATCTCACCGTTAT |
